# Bottom‐up effect of host protective symbionts on parasitoid diversity: Limited evidence from two field experiments

**DOI:** 10.1111/1365-2656.13650

**Published:** 2022-01-16

**Authors:** Karthik Sankar Narayan, Christoph Vorburger, Nina Hafer‐Hahmann

**Affiliations:** ^1^ Department of Aquatic Ecology Eawag Swiss Federal Institute of Aquatic Science and Technology Dübendorf Switzerland; ^2^ Institute of Integrative Biology ETH Zürich Zürich Switzerland

**Keywords:** aphids, bottom‐up effects, defensive symbiosis, diversity, field experiment, food web, parasitoid wasps, specificity

## Abstract

1. Protective symbionts can provide effective and specific protection to their hosts. This protection can differ between different symbiont strains with each strain providing protection against certain components of the parasite and pathogen community their host faces. Protective symbionts are especially well known from aphids where, among other functions, they provide protection against different parasitoid wasps. However, most of the evidence for this protection comes from laboratory experiments.
2. Our aim was to understand how consistent protection is across different symbiont strains under natural field conditions and whether symbiont diversity enhanced the species diversity of colonizing parasitoids, as could be expected from the specificity of their protection.
3. We used experimental colonies of the black bean aphid *Aphis fabae* to investigate symbiont‐conferred protection under natural field conditions over two seasons. Colonies differed only in their symbiont composition, carrying either no symbionts, a single strain of the protective symbiont *Hamiltonella defensa*, or a mixture of three *H*. *defensa* strains. These aphid colonies were exposed to natural parasitoid communities in the field. Subsequently, we determined the parasitoids hatched from each aphid colony.
4. The evidence for a protective effect of *H*. *defensa* was limited and inconsistent between years, and aphid colonies harbouring multiple symbiont strains did not support a more diverse parasitoid community. Instead, parasitoid diversity tended to be highest in the absence of *H*. *defensa*.
5. Symbiont‐conferred protection, although a strong and repeatable effect under laboratory conditions may not always cause the predicted bottom‐up effects under natural conditions in the field.

## Graphical abstract

Protective symbionts effectively protect their host against natural enemies. In aphids, such symbionts provide protection against parasitoid wasps. Given the specificity of this protection, we expect symbiont diversity to promote parasitoid diversity. However, in a field experiment exposing aphids to natural parasitoid communities such effects were limited.

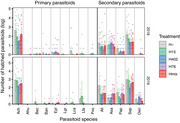

## 1 INTRODUCTION

Diversity at one trophic level can promote diversity at other trophic levels in a food web. There is comparative and experimental evidence that high species diversity in primary producers provides a more diverse resource base and thus promotes biodiversity across multiple levels of consumers (Scherber et al., 2010; Sobek et al., 2009). Much of the support for cascading diversity effects comes from biodiversity experiments manipulating the number and composition of plant species (reviewed by Siemann et al., 1998; Weisser et al., 2017). However, such effects are not restricted to species‐level diversity. Phenotypic variation resulting from genetic variation within plant species also affect diversity at higher trophic levels (Barbour et al., 2016; Crutsinger et al., 2006; Johnson et al., 2006).

Although less studied, similar bottom‐up effects can be expected for animal resources, for example on parasite diversity, since parasites are often highly specialized (the ‘host diversity begets parasite diversity hypothesis’, Johnson et al., 2016; Thieltges et al., 2011). Within‐species diversity in host susceptibility to parasites may arise from genetic variation for defence traits (e.g. Ebert et al., 1998; Sandrock et al., 2010; Smith et al., 1999), as well as from their association with symbionts that provide protection (Flórez et al., 2015; Haine, 2008). Such defensive symbioses include conditionally mutualistic associations like honeydew‐collecting ants defending sap‐sucking insects (Stadler & Dixon, 2005), relatively loose associations of animals with environmentally acquired ‘probiotic’ gut symbionts (Neish, 2009) and very tight associations with maternally transmitted microbes providing protection (Oliver & Moran, 2009). Heritable defensive symbionts can be seen as a second line of defence in addition to the host's intrinsic immune system. They provide effective and specific resistance against parasites and pathogens and thereby show some parallels to the immune systems of animals, such as the vertebrate major histocompatibility complex (MHC). Defensive symbionts should hence be subjected to similar evolutionary forces (Hafer & Vorburger, 2019). These have repeatedly resulted in the diversification of immune systems in response to diverse parasites and pathogens (Ghosh et al., 2011; Litman et al., 2007; Messier‐Solek et al., 2010). Similarly, protective symbionts and the parasites and pathogens they protect against are expected to drive each other's diversity (Hafer & Vorburger, 2019). Over ecological time frames, host populations possessing more diverse communities of protective symbionts are thus expected to support more diverse communities of parasites.

Protective symbionts are especially well‐studied in aphids, whom they protect against parasitoid wasps and pathogenic fungi (Guo et al., 2017; Oliver et al., 2014; Vorburger, 2014). These benefits are usually set off by fitness costs these symbionts impose in the absence of natural enemies (Cayetano et al., 2015; Heyworth & Ferrari, 2015; Oliver et al., 2008; Parker et al., 2017; Vorburger & Gouskov, 2011). As a consequence, secondary symbionts tend to occur at intermediate frequencies in nature (reviewed by Guo et al., 2017; McLean et al., 2016; Oliver et al., 2014; Zytynska & Weisser, 2016). Correlations between parasitoids and the symbiont communities of their aphid hosts observed in natural populations suggest that these frequencies may be partially driven by interactions between symbionts and parasitoids (Hafer‐Hahmann & Vorburger, 2021; Smith et al., 2015). However, more experimental approaches are required to establish the causal factors underlying these associations.

Probably the best studied symbiont when it comes to providing defences against parasitoid wasps is the gammaproteobacterium *Hamiltonella defensa* (Moran et al., 2005), a heritable endosymbiont of aphids. Protection by *H. defensa* differs between strains and is highly specific, with each symbiont conferring protection against certain parasitoid species (Cayetano & Vorburger, 2015; Leclair et al., 2016; Martinez et al., 2016; McLean & Godfray, 2015) or genotypes (Cayetano et al., 2015; Cayetano & Vorburger, 2013, 2015; Rouchet & Vorburger, 2012; Schmid et al., 2012; Vorburger & Rouchet, 2016), but not against others. The possession of *H*. *defensa* is highly beneficial in the presence of parasitoids. Parasitoids have been shown repeatedly to select for an increased prevalence of protective symbionts in aphid populations (Oliver et al., 2008; Rossbacher & Vorburger, 2020; Vorburger, 2014). Taking this one step further, two recent experiments could show that parasitoid diversity can be crucial in maintaining symbiont strain diversity (Hafer‐Hahmann & Vorburger, 2020; Rossbacher & Vorburger, 2020). Likewise, symbiont‐conferred protection has been shown to exert strong selection on parasitoids under laboratory condition, promoting the evolution of counteradaptations to overcome resistance mediated by *H*. *defensa* (Dennis et al., 2017; Dion et al., 2011) or even driving parasitoids to extinction (Käch et al., 2018). However, it remains unknown to which extent symbiont diversity can shape the composition and diversity of parasitoid communities.

Under natural conditions, the evidence for symbiont‐conferred protection and its benefits is less clear. In a field experiment, Rothacher et al. (2016) found that a single strain of *H*. *defensa* did indeed reduce parasitism rates of the black bean aphid *Aphis fabae*. Because the defence provided by *H*. *defensa* was unequally effective against different parasitoid species, the symbiont also altered parasitoid community composition and increased evenness by reducing the abundance of the dominant parasitoid species in protected aphids. Interestingly, the protection by *H*. *defensa* did not result in increased aphid population size in the presence of *H*. *defensa*, assumedly due the costs it imposes on aphid fitness in the absence of parasitoids (Rothacher et al., 2016). Similarly, Hrček et al. (2016) showed under field conditions that two symbionts, *H*. *defensa* and *Regiella insecticola*, protect pea aphids *Acyrthosiphon pisum* against parasitoids and pathogenic fungi, respectively, but again this did not result in overall benefits for the aphids since this protection was offset by other causes of mortality. By contrast, a more recent study failed to find any effect of *H*. *defensa* in protecting *Aphis craccivora* against the local parasitoid community (Lenhart & White, 2017).

Here, we conducted a field experiment with black bean aphids *Aphis fabae*, exposing *H*. *defensa*‐free and *H*. *defensa*‐infected populations to test for bottom‐up effects of *H*. *defensa* on naturally colonizing parasitoid communities. In contrast to previous experiments, we worked with three different strains of *H*. *defensa*, creating different singly infected populations as well as populations possessing multiple strains by mixing the three singly infected lines. This allowed us to address the following questions: (1) How effective is symbiont‐conferred protection in the field and is it comparable for different strains of *H*. *defensa*? (2) Do more diverse symbiont communities lead to more diverse parasitoid communities, that is does symbiont diversity influence parasitoid diversity? Surprisingly, the evidence for protection and differences between *H*. *defensa* strains was very limited in our study.

## 2 MATERIALS AND METHODS

### 2.1 Experimental set‐up

Experiments consisted of exposing black bean aphids *Aphis fabae fabae* on their broad bean *Vicia faba* host to natural parasitoid communities over two seasons in 2018 and in 2019. In order to test for differences among protective symbionts without any confounding effects of host genotype, we used an uninfected and three *H*. *defensa*‐infected lines of only one clone each year. We used clone A06‐405 in 2018 and a different clone, A06‐407, in 2019. The lines carrying *H*. *defensa* were generated by microinjection of haemolymph from donor clones infected with three different *H*. *defensa* strains, H15, H76 and H402 (Cayetano et al., 2015), which resulted in stable, heritable infections. The lines have since been kept in the laboratory at 18–20°C and a 16/8‐hr light/dark regime, which ensures continuous parthenogenetic reproduction. Using the four available lines, we set up five different treatments each year: aphids without *H*. *defensa* (H‐), aphids infected with H15, H76 or H402 and aphids comprising an equal mixture of all three *H*. *defensa*‐containing lines: Hmix. While the aim was the same in both years, the exact set‐ups differed.

In 2018, the exposures were carried out in three consecutive temporal blocks with five replicates per treatment in each block. A replicate consisted of a square plastic plant pot (34 × 34 × 32 cm, with water reservoir) containing three bean plants carrying aphid colonies of one treatment (H‐, H15, H76, H402 or Hmix). Replicates were arranged at random positions in a grid with about 1 m distance between pots inside a fenced area of the Eawag campus in Dübendorf, Switzerland. Plants were grown from seeds in the laboratory. When they were 3 weeks old, we inoculated them with nine adult aphids of the appropriate lines and allowed aphids to reproduce for another week. Then we planted three thus inoculated plants into each pot at their outdoor location. All plants within a pot belonged to the same treatment and aphids could move freely between plants within a pot. The area surrounding the experimental site contained diverse natural vegetation which harboured various species of aphids, natural enemies of aphids and ants that frequently tend black bean aphids. The first exposure started on 14 June 2018 and lasted for 19 days, the second and third started on 5 and 26 July, respectively, and lasted for 14 days each. At the end of each exposure, we collected all plants and associated insects (see sampling and measurements below).

In 2019, the experiment comprised six replicates of each treatment (30 pots in total) that were set up with one replicate per treatment in each of six spatial blocks spread out over six different locations across the Eawag campus. These sites varied somewhat in the surrounding vegetation, ranging from relatively open meadow to sites adjacent to shrubs or hedges. One location was the same as the one used in the 2018 experiment. Rather than collecting all plants and insects after a period of exposure, sampling took place continuously in 2019 (see Rothacher et al., 2016 for a similar set‐up). We started the experiment by planting the outdoor pots with four 4‐week old bean plants that had been inoculated with 12 aphids of the appropriate treatment for 10 days. After 2 weeks in the field, we collected one plant per pot (see sampling below) and replaced it with a new plant inoculated with the same initial number and composition of aphids. The same procedure was repeated weekly for another 12 weeks, always harvesting the oldest plants after all of the first four plants had been harvested. However, some adjustments had to be made during the course of the experiment. The number of adult aphids used to inoculate the plants was reduced from 12 (round 1–9) to 9 (round 10–13) because the size of colonies that had developed when we put out the plants was sometimes so large that it adversely impacted the plants. In the fifth sampling round, we harvested two plants and replaced them with one only to reduce the exposure time in the field from 4 to 3 weeks. We did not provide a replacement plant in the week prior to final sampling and harvested the two last plants on consecutive days so that one of them had only been in the field for 2 weeks. In total, this resulted in the harvesting of 13 plants per pot. Heatwaves in June resulted in the death of some plants. These were immediately (within 24 hr) replaced with new plants of approximately the same age but without any aphids. The continued addition of new aphids ensured that treatment differences in aphid composition persisted throughout the experiment, even though migration between pots or influx of wild aphids would have been possible. Indeed, a test towards the end of the experiment confirmed that mostly aphids remained in their appropriate treatment (see Supporting Information, test for migration).

We reduced access of snails and slugs to pots and removed any snail or slug we found, but otherwise pots were freely accessible to ants, parasitoids, predators and other animals. Plants were watered whenever necessary, albeit due to the water reservoirs within the pots this was rarely the case.

No ethical approval was required for this work.

### 2.2 Sampling and measurements

Sampling (at the end of each block in 2018, weekly in 2019) took place by gently placing a cellophane bag over each plant prior to cutting the plant and immediately sealing the bag to ensure that all animals located on the plant at that particular moment were trapped. We measured the length of each plant and counted the number of aphids either by counting aphids in groups of roughly five individuals (2018) or counting their exact number (2019). We also counted the number of mummies (dead aphids that were parasitized successfully), irrespective of whether or not the parasitoid had already hatched. Unhatched mummies (usually the large majority of mummies) were collected in insect dishes for hatching and subsequent identification. We additionally kept the plants in the cellophane bags to be able to catch and determine any parasitoids that would hatch from mummies that only formed after collection. Lastly, we counted all ants and predators. For predators, we recorded which order they belonged to and which stage they were in (i.e. egg, larvae, pupae or adult).

After they had hatched, we determined parasitoids under a stereo microscope using several keys (Hullé et al., 2020; Japoshvili & Abrantes, 2006; Japoshvili & Karaca, 2009; Kavallieratos et al., 2013; Tomanović et al., 2018). We identified primary parasitoids to the species level and secondary parasitoids at least to the genus level. See Table S1 for a complete list of all parasitoids identified.

### 2.3 Statistical analysis

All analysis and plot generation (package ggplot2; Wickham, 2016) took place in R, version 3.6.1 (R Core Team, 2019). We used linear mixed effect models (package lme4; Bates et al., 2015) to analyse the effect of aphid endosymbiont treatments on the number of mummies, mummification rate (the number of mummies divided by the number of mummies and live aphids within each replicate), the number of hatched parasitoids, the number of aphids, plant size and parasitoid diversity (species number and Shannon index for all parasitoids and for primary parasitoids only). To analyse mummification, we excluded samples where neither aphids nor mummies were found due to predation or aphid emigration. Parasitoid species number and Shannon diversity were calculated with *vegan* (Oksanen et al., 2019). In addition to diversity estimates based on raw count data, we obtained rarefied species richness and Shannon index through function *estimateD* in *iNEXT* (Hsieh et al., 2016) using only samples with at least five parasitoids and a resample size of 5. We did this for primary parasitoids and for all parasitoids combined (primary and secondary). Prior to analysis, we transformed response variables using *TransformTukey* from the package rcompanion (Mangiafico, 2019) to ensure that model assumptions were met. We analysed data from both years separately. For 2018, we first calculated data over all plants from each pot and block which we used in subsequent analysis and included block as random effect. For 2019, we used individual data from each round (i.e. sampling event) for aphid number, mummy number, mummification rate and plant size, and we included sampling round, plot and pot (nested within plot) as random effects. For the number of hatched parasitoids and parasitoid diversity in 2019, we calculated summed numbers over all rounds for each pot and included plot as random effect.

In each case, we built a linear mixed model including the contrasts *H*. *defensa* presence versus absence, *H*. *defensa* diversity (3 vs. 1 different haplotype) and *H*. *defensa* strain (among strains; comparison against H402) in that order as fixed effects to partition variance among them. We included aphid number as covariate and first factor in the formula when analysing mummy number, mummification rate, number of hatched parasitoids and plant size. Each model was followed with a type I analysis of variance using Satterthwaite's method to obtain *p*‐values.

To analyse parasitoid species composition, we used parasitoids summarized over all plants for each block for 2018 and summarized all parasitoids per pot over all rounds for 2019 to obtain a single species matrix for each independent replicate. To test whether parasitoid community composition depended on treatment, we conducted a distance‐based redundancy analysis (dbRDA) from the package vegan (Oksanen et al., 2019). We used rank correlations between dissimilarity indices and gradient separation to select the best dissimilarity index to use for subsequent analysis. We obtained significance values using permutation tests with 999 permutations.

## 3 RESULTS

**FIGURE 1:**
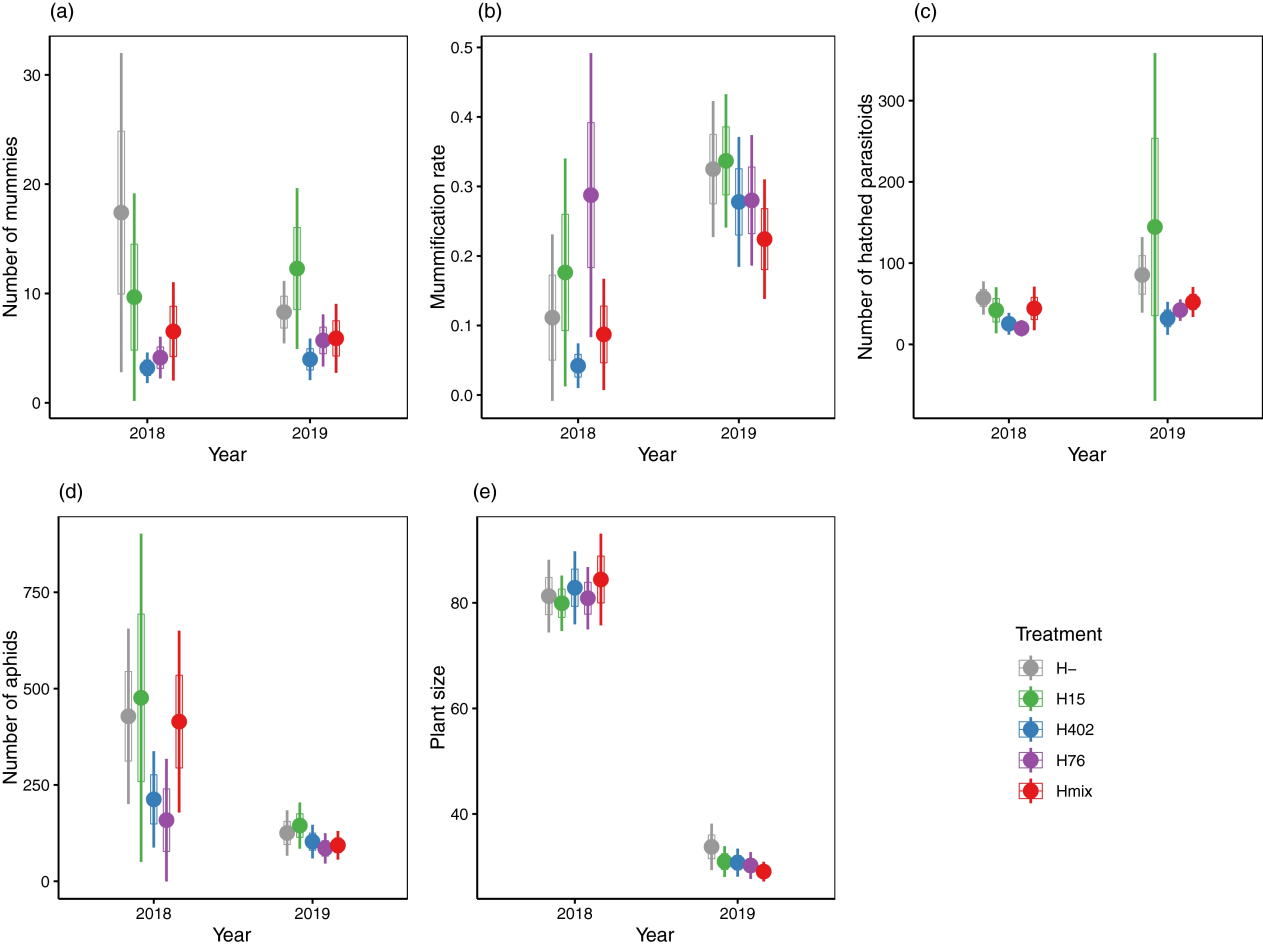
Mummy numbers (a), mummification rate (b), number of hatched parasitoids (c), aphid number (d) and plant size (e) by treatment. Please note that in 2018 the experiment was carried out in three consecutive rounds with five replicates of each treatment per round, whereas in 2019, the experiment was arranged in six spatial blocks with one replicate per treatment that was sampled repeatedly over time. This was accounted for in the analysis but not for plotting. Please refer to Figures S1 and S2 for more detailed plots. Mummification rate was calculated for each individual replicate prior to calculating mean and CI, giving equal weight to replicates with different numbers of individuals remaining on the plants. This explains why mean mummification rates are higher than what mean numbers of mummies and aphids would suggest. Error bars represent 95% CI, boxes represent SE. H‐: *H*. *defensa*‐free aphids, H15: Aphids carrying *H*. *defensa* haplotype 15, H402: Aphids carrying *H*. *defensa* haplotype 402, H76: Aphids carrying *H*. *defensa* haplotype 76 and Hmix: Aphids carrying *H*. *defensa* of different haplotypes. Please note that data are for three plants per sample in 2018 and for a single plant per sample in 2019. Number of hatched parasitoid (c) is summed over all rounds for each pot in 2019 [Correction added on 7‐July‐2022, after first online publication: In the PDF version, Figure 1 has been replaced with the correct figure.]

In both years, the treatments with the highest total number of mummies were H‐ and H15. In 2018, *H*. *defensa* appeared to provide significant protection in that significantly more mummies and hatched parasitoids were obtained from the *H*. *defensa*‐free aphids compared to the average of all *H*. *defensa*‐infected treatments (Figure 1a,c; Table 1; Table S2). However, this was at least partially related to a somewhat higher number of aphids on the plants of this treatment (marginally non‐significant, Table 1; Figure 1d), such that the proportion of mummified aphids (mummification rate) did not differ significantly between *H*. *defensa*‐free and *H*. *defensa*‐infected aphids (Figure 1b; Table 1). The number of different *H*. *defensa* strains (1 vs. 3) did not have a significant effect on any of these responses, nor were there significant differences among the three treatments with single *H*. *defensa* strains (Figure 1a,b,d; Table 1; Table S2), albeit *H*. *defensa* strain showed a non‐significant trend to affect mummification rate in 2018 (Figure 1b; Table 1; Table S2), which was highest with haplotype H402 and lowest with H76 in this year. None of the treatment differences observed in 2018 were significant in the 2019 experiment with the exception of the number of hatched parasitoids, which differed among the three treatments with one *H*. *defensa* strain. It was highest in the H15 treatment, but the variation among replicates was enormous (Figure 1c; Figures S1 and S2; Table 1; Table S2). Plant size was not affected by our treatments in either year (Figure 1e; Table 1; Table S2).

**TABLE 1:** Outcome of type I analysis of variance for different responses. Factors were added to the model in the order specified in the table. Significant *p*‐values for treatments have been highlighted in bold, marginally significant ones in italic. Where appropriate, we accounted for the number of aphids as a covariate

Factor	2018			2019		
	*df*	*F*	*p*	*df*	*F*	*p*
Mummy number						
Number of aphids	1,63	16.31	0.0001	1,364	15.95	0.0001
*H. defensa* infected versus uninfected	1,67	4.33	**0.0414**	1,19	2.05	0.1680
*H. defensa* diversity	1,67	0.07	0.7919	1,19	0.47	0.4991
*H. defensa* strain	2,67	0.44	0.6444	2,19	2.48	0.1096
Mummification rate						
Number of aphids	1,68	4.60	0.0355	1,277	22.42	0.0000
*H. defensa* infected versus uninfected	1,68	0.19	0.6614	1,20	1.57	0.2249
*H. defensa* diversity	1,68	0.77	0.3845	1,21	2.93	0.1013
*H. defensa* strain	2,68	2.89	*0.0622*	2,21	2.34	0.1210
Number of hatched parasitoids						
Number of aphids	1,52	65.66	0.0000	1,22	35.32	0.0000
*H. defensa* infected versus uninfected	1,67	6.98	**0.0102**	1,19	2.56	0.1256
*H. defensa* diversity	1,67	0.07	0.7894	1,19	2.64	0.1204
*H. defensa* strain	2,68	0.40	0.6692	2,19	4.25	**0.0296**
Aphid number						
*H. defensa* infected versus uninfected	1,68	3.60	*0.0619*	1,20	0.00	0.9802
*H. defensa* diversity	1,68	3.36	*0.0713*	1,20	0.03	0.8735
*H. defensa* strain	2,68	1.56	0.2178	2,20	0.33	0.7216
Plant size						
Number of aphids	1,59	1.33	0.2526	1,289	0.53	0.4653
*H. defensa* infected versus uninfected	1,67	0.01	0.9289	1,20	2.09	0.1637
*H. defensa* diversity	1,67	0.82	0.3698	1,20	0.39	0.5409
*H. defensa* strain	2,67	0.12	0.8915	2,20	0.05	0.9512

**FIGURE 2:**
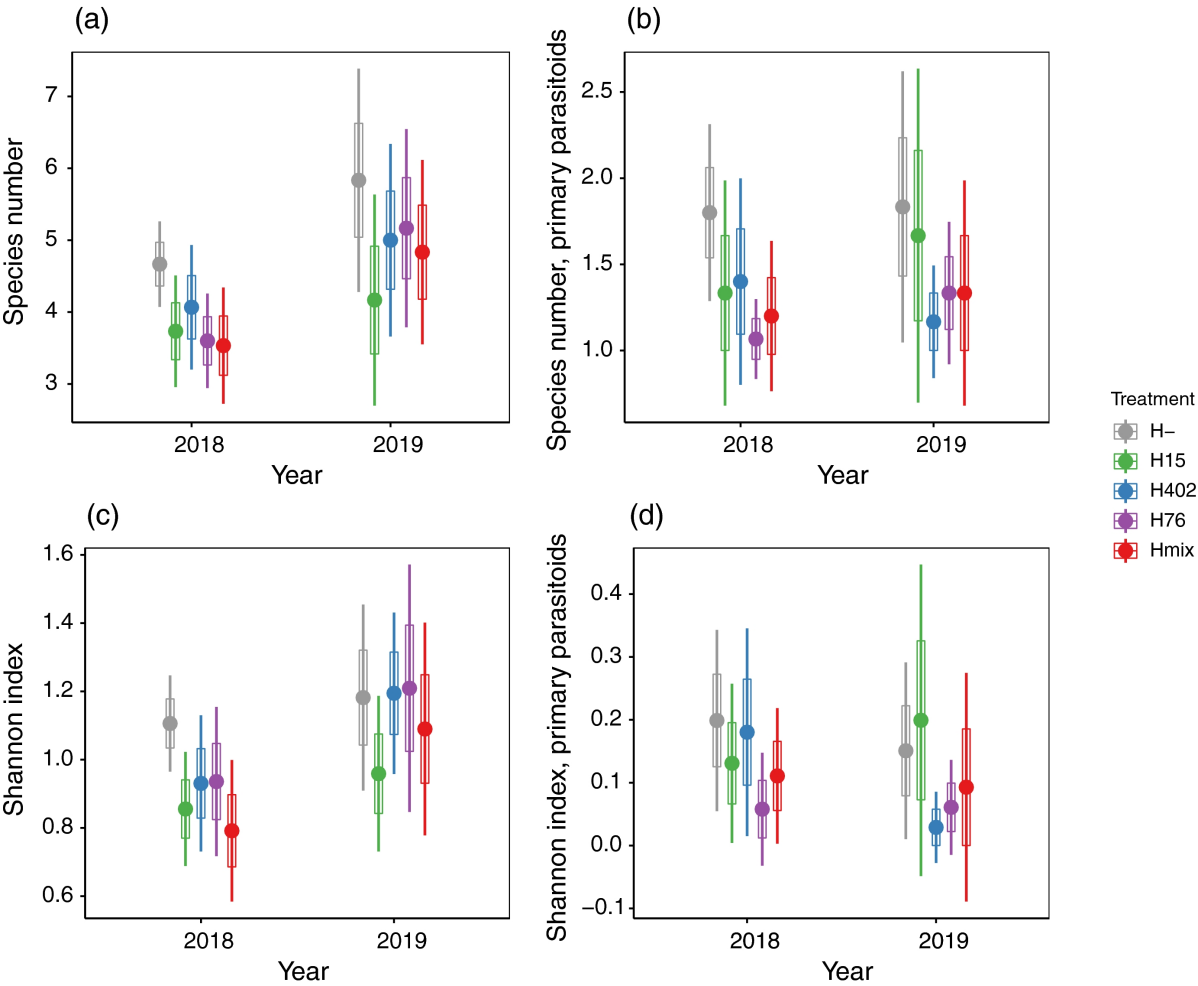
Species number of all (a) and of primary parasitoids (b) and Shannon diversity for all (c) and primary parasitoids only (d) by treatment. Please note that in 2018 the experiment was carried out in three consecutive rounds with five replicates of each treatment per round. By contrast, in 2019, the experiment was arranged in six spatial blocks with one replicate per treatment that was sampled repeatedly over time and results were summed over all sampling events prior to plotting and analysis. This was accounted for in the analysis but not for plotting. Error bars represent 95% CI, boxes represent SE. H‐: *H*. *defensa*‐free aphids, H15: Aphids carrying *H*. *defensa* haplotype 15, H402: Aphids carrying *H*. *defensa* haplotype 402, H76: Aphids carrying *H*. *defensa* haplotype 76 and Hmix: Aphids carrying *H*. *defensa* of different haplotypes. Please refer to Figure S3 for rarefied diversity estimates

In both years, the total number of parasitoid species obtained from aphids without *H*. *defensa* was significantly higher than in all treatments with *H*. *defensa*‐infected aphids (Figure 2a; Table 2; Table S3). Similarly, Shannon diversity over all parasitoid species was significantly higher in the H‐ treatment than in all other treatments in 2018, but not in 2019 (Figure 2c; Table 2; Table S3). These patterns were similar when looking at primary parasitoids only (Figure 2b,d; Table 2; Table S3).

**TABLE 2:** Outcome of type I analysis of variance for different measures of parasitoid diversity. Factors were added to the model in the order specified in the table. Significant *p*‐values for treatments have been highlighted in bold, marginally significant ones in italic

Factor	2018			2019		
	*df*	*F*	*p*	*df*	*F*	*p*
Species number						
*H. defensa* infected versus uninfected	1,70	4.99	**0.0288**	1,20	6.81	**0.0168**
*H. defensa* diversity	1,70	0.40	0.5308	1,20	0.00	0.9650
*H. defensa* strain	2,70	0.34	0.7130	2,20	1.30	0.2959
Species number, primary parasitoids						
*H. defensa* infected versus uninfected	1,70	4.18	**0.0447**	1,20	1.80	0.1951
*H. defensa* diversity	1,70	0.05	0.8237	1,20	0.05	0.8221
*H. defensa* strain	2,70	0.15	0.8609	2,20	0.45	0.6436
Shannon index						
*H. defensa* infected versus uninfected	1,68	4.75	**0.0327**	1,20	0.67	0.4235
*H. defensa* diversity	1,68	1.11	0.2956	1,20	0.13	0.7255
*H. defensa* strain	2,68	0.29	0.7513	2,20	3.91	**0.0369**
Shannon index, primary parasitoids						
*H. defensa* infected versus uninfected	1,70	1.80	0.1841	1,20	0.81	0.3798
*H. defensa* diversity	1,70	0.02	0.8809	1,20	0.04	0.8427
*H. defensa* strain	2,70	0.85	0.4302	2,20	0.74	0.4895
Rarefied species number						
*H. defensa* infected versus uninfected	1,68	3.04	*0.0859*	1,20	0.38	0.5430
*H. defensa* diversity	1,68	1.33	0.2524	1,20	0.23	0.6383
*H. defensa* strain	2,68	0.50	0.6107	2,20	3.72	**0.0424**
Rarefied species number, primary parasitoids						
*H. defensa* infected versus uninfected	1,70	2.57	0.113	1,20	0.91	0.3519
*H. defensa* diversity	1,70	0.14	0.710	1,20	0.03	0.8599
*H. defensa* strain	2,70	0.22	0.806	2,20	0.73	0.4928
Rarefied Shannon index						
*H. defensa* infected versus uninfected	1,68	2.37	0.1284	1,20	0.12	0.7291
*H. defensa* diversity	1,68	1.35	0.2500	1,20	0.23	0.6353
*H. defensa* strain	2,68	0.44	0.6464	2,20	3.25	*0.0600*
Rarefied Shannon index, primary parasitoids						
*H. defensa* infected versus uninfected	1,70	1.74	0.1920	1,20	0.80	0.3793
*H. defensa* diversity	1,70	0.01	0.9142	1,20	0.06	0.8078
*H. defensa* strain	2,70	0.85	0.4297	2,20	0.81	0.4541

We cannot rule out that some of our findings may have been affected by differences in sample size (i.e. the number of hatched parasitoids obtained), albeit this seems unlikely considering that the treatment that showed the lowest diversity when using all parasitoids, H15, showed the highest number of hatched parasitoids. Nevertheless, any significant effects disappeared or became marginally non‐significant when using rarefied diversity estimates (Figure S3; Table 2; Table S3), although we observed a significant effect for *H*. *defensa* strain on rarefied species number in 2019 (*p* = 0.042), which again seems to have been driven by a small number of species for H15 (Figure S3a).

**FIGURE 3:**
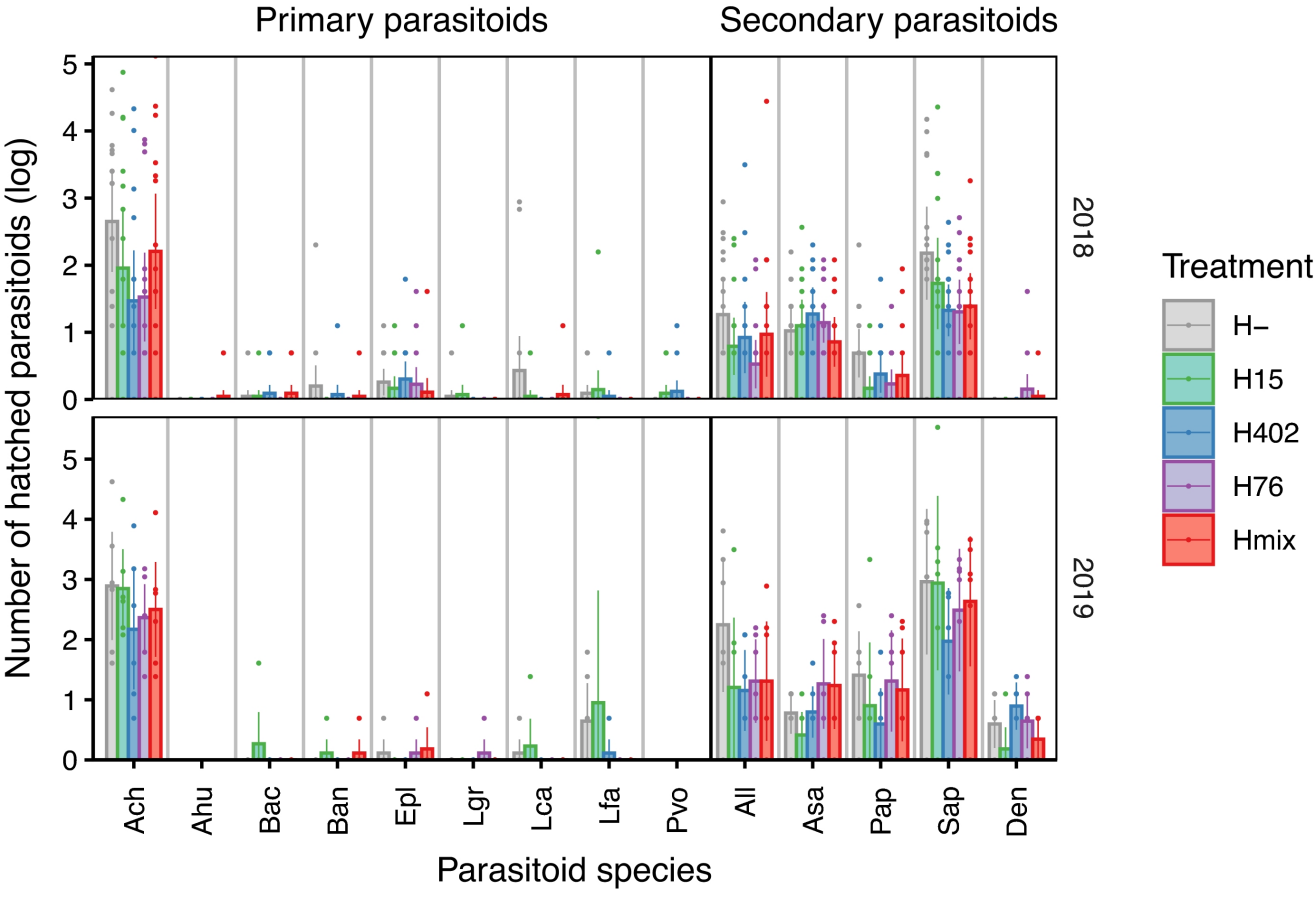
Number of hatched parasitoids of each parasitoid species. Error bars represent 95% CI. H‐: *H. defensa*‐free aphids, H15: Aphids carrying *H. defensa* haplotype 15, H402: Aphids carrying *H. defensa* haplotype 402, H76: Aphids carrying *H. defensa* haplotype 76 and Hmix: Aphids carrying *H. defensa* of different haplotypes. Ach: *Aphelinus chaonia*, Ahu: *A. humilis*, Bac: *Binodoxys acalaphae*, Ban: *B. angelicae*, Epl: *Ephedrus plagiator*, Lgr: *Lipolexis gracilis*, Lca: *Lysiphlebus cardui*, Lfab: *L. fabarum*, Pva: *Praon volucre*, All: *Alloxysta* spp., Asa: *Asaphes* spp., Pap: *Prachyneuron aphidis*, Sap: *Syrphophagus aphidivorus*, Den: *Dendrocerus* spp

Treatment had no clear effect on either total parasitoid composition (2018: *F*_4,68_ = 1.40, *p* = 0.123; 2019: *F*_4,24_ = 1.18, *p* = 0.242; Figure 3) or primary parasitoid composition (2018: *F*_4,58_ = 1.22, *p* = 0.254; 2019: *F*_4,24_ = 0.711, *p* = 0.842; Figure 3) in either year. Nevertheless, treatment explained 8.03% (all parasitoids, 2018), 17.43% (all parasitoids, 2019), 7.02% (primary parasitoids, 2018) or 11.51% (primary parasitoids, 2019) of variance in parasitoid communities.

## 4 DISCUSSION

Intraspecific variation can strongly affect community structure in food webs, with effects of a magnitude comparable to those of interspecific diversity (Hahn et al., 2017; Raffard et al., 2019). Here, we tested whether intraspecific differences in an aphid host, mediated not by the species' genotype but by the genotype of its heritable protective symbiont, could exert bottom‐up effects on subsequent trophic levels, that is primary and secondary parasitoids (McLean et al., 2016). The experiment was motivated by the fact that symbiont‐conferred resistance in aphids is highly specific, with different strains of *H*. *defensa* providing unequal protection against different parasitoid species and or different genotypes of the same parasitoid species (Cayetano et al., 2015; Cayetano & Vorburger, 2013, 2015; Leclair et al., 2016; McLean & Godfray, 2015; Rouchet & Vorburger, 2012; Schmid et al., 2012). Hence, we predicted that higher symbiont diversity should promote higher parasitoid diversity (see Hafer & Vorburger, 2019), yet we did not observe any such effect in our field experiments. There was not even any strong evidence that the species composition of emerging parasitoids was influenced significantly by the different *H*. *defensa* strains (Figure 3). Only in 2019 did we observe an effect of *H*. *defensa* strain in that rarefied species number was lowest in strain H15, the least protective strain in our experiment. Additionally, Shannon diversity (in 2018 only) and the number of parasitoid species (both years) were higher in *H*. *defensa*‐free aphids, which were expected to be more permissive hosts to begin with. The relatively low rates of parasitism have certainly limited the opportunity to detect responses in terms of species composition and diversity, and it was probably further reduced by a very high incidence of secondary parasitoids (Figure 3), which may well have erased any symbiont effects there may have been on primary parasitoids. We also suspect that potential effects of symbiont diversity on parasitoid diversity may have been obscured by very high aphid mortality that was unrelated to parasitism. We often obtained low aphid counts and even observed complete losses of aphids on individual plants. Aphid mortality seems to have been driven especially by a high abundance of predators. In line with previous findings (Costopoulos et al., 2014; Kovacs et al., 2017), these predators seem to have preyed indiscriminately of *H*. *defensa* in our experiments (see, Effect of treatment on further variables in the Supporting Information Results). This shows that even clear laboratory results on the species‐specificity of protection (e.g. Asplen et al., 2014; Cayetano & Vorburger, 2015; McLean & Godfray, 2015) do not necessarily predict the importance of such effects in the natural environment, where other sources of mortality come into play.

Also our more straightforward prediction that parasitism should be reduced in *H*. *defensa*‐protected aphid populations was not well‐supported, despite working with strains of *H*. *defensa* that are known to confer resistance against several parasitoids under laboratory conditions. Even though in both years we did obtain the highest number of parasitoid wasp individuals out of *H*. *defensa*‐free aphids and aphids infected with H15, the least protective of the three symbiont strains (Cayetano et al., 2015; Schmid et al., 2012), the rate of parasitism did not differ significantly among treatments. This is in contrast to an earlier study by Rothacher et al. (2016), who found clear evidence for protection using a similar approach, but only compared between *H*. *defensa*‐free aphids and aphids infected with a single *H*. *defensa* strain, H402. We know that the symbionts have not lost their protective phenotype between these experiments. Most likely, the differences are explicable by differences in parasitoid occurrence. During the field experiment of Rothacher et al. (2016), the parasitoid community was dominated by a single species, *Lysiphlebus fabarum*, normally *A*. *fabae*'s most frequent parasitoid (Starý, 2006; Vorburger & Rouchet, 2016), against which *H*. *defensa* provides effective protection (Schmid et al., 2012; Vorburger et al., 2009). Unexpectedly, *Lysiphlebus* spp. was very rare in our experiments, which may well‐explain the seeming lack of protection we observed. In 2019, when *L*. *fabarum* was abundant enough to conduct meaningful comparisons, it was indeed more frequent in the absence than in the presence of *H*. *defensa* (see, Effect of treatment on further variables in the Supporting Information Results), supporting the idea that we would have observed protection if *L*. *fabarum* had been more abundant in our experiments. The only primary parasitoid that occurred at an appreciable frequency in our experiment was *Aphelinus chaonia*. For this species, it was more difficult to predict the effect of *H*. *defensa*, as there are few prior studies to draw on. A single laboratory experiment with *A*. *fabae* suggested that *H*. *defensa* offers little protection against *A*. *chaonia* (Cayetano & Vorburger, 2015), but the field experiment by Rothacher et al. (2016) actually obtained significantly fewer *A*. *chaonia* from *H*. *defensa*‐infected than from *H*. *defensa*‐free aphids. Results from other species of *Aphelinus* are also mixed. Some strains of *H*. *defensa* do protect pea aphids against *A*. *abdominalis* (McLean & Godfray, 2015), while there is no evidence for *H*. *defensa*‐mediated resistance to *A*. *atriciplis* and *A*. *glycinis* (Hopper et al., 2018). In our field experiments, parasitism by *A*. *chaonia* showed a slight and non‐significant tendency to be deterred by *H*. *defensa* (see, Effect of treatment on further variables in the Supporting Information Results). The results on protection overall certainly give reason for caution in that even strong evidence for protection from the laboratory may not predict its ecological importance in the field, and in that results from one field experiment may not readily be generalized when conditions vary widely among locations or years (e.g. the unusually low abundance of *L*. *fabarum* in 2018/2019).

Can any general lessons be learnt from our largely negative results? We were certainly surprised that differences in a trait that is clearly relevant ecologically—resistance to parasitoids—did not have a larger effect on the ensuing trophic networks, at least not in these experiments. In laboratory cage experiments, the effect of defensive symbionts on parasitism are unambiguous and strong (Käch et al., 2018; Oliver et al., 2008), and the role of interaction specificity in maintaining variation is demonstrable (Hafer‐Hahmann & Vorburger, 2020; Rossbacher & Vorburger, 2020). There is also evidence from slightly more complex aphid–parasitoid–symbiont communities maintained in mesocosms that the presence of a protective symbiont can destabilize an experimental parasitoid community so strongly that local extinctions ensue (Sanders et al., 2016). However, laboratory or mesocosm experiments isolate a subset of interacting species from their surrounding community, which is at the same time an advantage and a limitation. They are better suited for a proof of principle that diversity at one trophic level can propagate to other trophic levels in a food web, but they tell us less about the actual importance of such effects in more complex natural communities. It is known that bottom‐up and top‐down effects are also affected by the surrounding community and its diversity (Cao et al., 2018; Crawford & Rudgers, 2013). More specifically, aphid species‐level diversity can enhance parasitoid diversity (Petermann et al., 2010) and plant diversity can enhance aphid symbiont diversity (Zytynska et al., 2016). We did not investigate plant or aphid communities surrounding our experimental plots, but it seems plausible that the diversity of adjacent plants, aphids and parasitoids, which form part of the same food web, play an important role in influencing the parasitoid community of any particular aphid colony. This will make the outcome of field experiments highly contingent on the immediate surroundings, requiring repetition of such experiments in different environments for generalities to emerge.

That being said, there are of course studies demonstrating bottom‐up effects of intraspecific diversity on field communities. Barbour et al. (2016), for example, observed that plant genetic variation strongly increased food web complexity by increasing the diversity of gall‐inducing insects and decreasing the level of specialization in their parasitoids. Similarly, plant genetic diversity can affect arthropod communities more generally (Crawford & Rudgers, 2013; Crutsinger et al., 2006; Müller et al., 2018). For the intraspecific variation induced by the infection with resistance‐conferring symbionts, our field experiments with aphids failed to produce conclusive evidence of bottom‐up effects on parasitoid communities, despite suggestive results obtained under laboratory conditions.

## CONFLICT OF INTEREST

The authors have no conflict of interest to report.

## AUTHORS' CONTRIBUTIONS

C.V. and N.H.‐H. designed the experiment; K.S.N. and N.H.‐H. performed the experiments with help from C.V.; N.H.‐H. analysed the data and C.V. contributed to data analysis; N.H.‐H. and C.V. wrote the manuscript with input from K.S.N. All authors approved the manuscript for publication.

## Supporting information

Fig S1‐S3

Table S1‐S3

Supplementary Material

Supplementary Material

## Data Availability

Data are available at Dryad Digital Repository https://doi.org/10.5061/dryad.1ns1rn8vr (Narayan et al., 2021).
