# Supplementary material for: Bottom‐up effect of host protective symbionts on parasitoid diversity: Limited evidence from two field experiments
Source: J Anim Ecol. 2022 Jan 16;91(3):643–54. doi: 10.1111/1365-2656.13650 (PMC9306599; doi:10.1111/1365-2656.13650)
Supplement: Supplementary file 1 — Fig S1‐S3 [file JANE-91-643-s001.docx]

# **Supplementary Figures**

## **Figure S1**

**Figure S1: Mummy number (A), mummification rate (B), number of hatched parasitoids (C), aphid number (D), and plant size (E) per block for 2018.** Small dots represent measurements per plant, large dots means within each pot, error bars represent 95% CI for each pot. H-: *H. defensa* free aphids, H15: Aphids carrying *H. defensa* haplotype 15, H402: Aphids carrying *H. defensa* haplotype 402, H76: Aphids carrying *H. defensa* haplotype 76, Hmix: Aphids carrying *H. defensa* of different haplotypes.

## **Figure S2**

Figure S2: Mummy number (A), mummification rate (B), number of hatched parasitoids (C), aphid number (D), and plant size (E) per plot (rows) over rounds (time) for 2019. H-: *H. defensa* free aphids, H15: Aphids carrying *H. defensa* haplotype 15, H402: Aphids carrying *H. defensa* haplotype 402, H76: Aphids carrying *H. defensa* haplotype 76, Hmix: Aphids carrying *H. defensa* of different haplotypes.

Figure S3

Figure S3: Rarefied diversity (1 vs. 3 strains) estimates for species number of all (A) and of primary parasitoids (B) and shannon diversity (1 vs. 3 strains) for all (C) and primary parasitoids only (D) by treatment. Error bars represent 95% CI, boxes represent SE. H-: *H. defensa* free aphids, H15: Aphids carrying *H. defensa* haplotype 15, H402: Aphids carrying *H. defensa* haplotype 402, H76: Aphids carrying *H. defensa* haplotype 76, Hmix: Aphids carrying *H. defensa* of different haplotypes.
