# Supplementary material for: Bottom‐up effect of host protective symbionts on parasitoid diversity: Limited evidence from two field experiments
Source: J Anim Ecol. 2022 Jan 16;91(3):643–54. doi: 10.1111/1365-2656.13650 (PMC9306599; doi:10.1111/1365-2656.13650)
Supplement: Supplementary file 2 — Table S1‐S3 [file JANE-91-643-s004.docx]

# **Supplementary Tables**

## **Table S1**

**Table S1: Parasitoids collected and identified in 2018 and 2019.**

|  | | **Species** | **Number, 2018** | **Number, 2019** |
| --- | --- | --- | --- | --- |
| **Primary parasitoids** | *Aphelinus chaonia* | | 1526 | 585 |
|  | *A. humilis* | | 1 |  |
|  | *Binodoxys acalephae* | | 6 | 4 |
|  | *B. angelicae* | | 13 | 2 |
|  | *Ephedrus plagiator* | | 30 | 4 |
|  | *Lipolexis gracilis* | | 3 | 1 |
|  | *Lysiphlebus cardui* | | 38 | 4 |
|  | *L. fabarum* | | 11 | 311 |
|  | *Praeon volucre* | | 5 |  |
| **Secondary parasitoids** | *Alloxysta* spp. | | 290 | 216 |
|  | *Asaphes* spp. | | 204 | 67 |
|  | *Dendrocerus* spp. | | 6 | 28 |
|  | *Pachyneuron aphidis* | | 58 | 115 |
|  | *Syrphophagus aphidivorus* | | 637 | 801 |

## Table S2

**Table S2: Summary of linear mixed models for different responses using treatments as fixed effect and block (2018) or plant nested within block and round (2019) as random effects.** In order to obtain coefficients, we used three separate models, one including only *H. defensa* presence versus absence to estimate its effect (and aphid Number where appropriate), one additionally including, *H. defensa* diversity (3 versus 1 different haplotypes) to determine the effect of diversity, and one additionally including *H. defensa* strain (among strains; comparison against H402) to obtain estimates for the effect of strain identity. Residuals, Number of aphids, and Intercepts were obtained from the final model.

**Random effects:**

| **Response** | **Year** |  | **Group** | **Variance** | **sd** |
| --- | --- | --- | --- | --- | --- |
| **Mummy number** | 18 |  | Block | 0.075 | 0.274 |
|  | 18 |  | Residual | 0.511 | 0.715 |
|  | 19 |  | Pot:plot | 0.039 | 0.197 |
|  | 19 |  | Round | 0.186 | 0.431 |
|  | 19 |  | Plot | 0.149 | 0.386 |
|  | 19 |  | Residual | 0.625 | 0.790 |
| **Mummification rate** | 18 |  | Block | <0.001 | <0.001 |
|  | 18 |  | Residual | 0.069 | 0.263 |
|  | 19 |  | Pot:plot | 0.002 | 0.043 |
|  | 19 |  | Round | 0.018 | 0.135 |
|  | 19 |  | Plot | 0.020 | 0.143 |
|  | 19 |  | Residual | 0.088 | 0.296 |
| **Number of hatched parasitoids** | 18 |  | Block | <0.001 | 0.006 |
|  | 18 |  | Residual | 0.001 | 0.023 |
|  | 19 |  | Plot | <0.001 | 0.014 |
|  | 19 |  | Residual | <0.001 | 0.010 |
| **Number of aphids** | 18 |  | Block | 1.270 | 1.127 |
|  | 18 |  | Residual | 1.953 | 1.397 |
|  | 19 |  | Pot:plot | 0.132 | 0.363 |
|  | 19 |  | Round | 0.554 | 0.744 |
|  | 19 |  | Plot | 0.318 | 0.564 |
|  | 19 |  | Residual | 1.227 | 1.108 |
| **Plant size** | 18 |  | Block | 0.285 | 0.534 |
|  | 18 |  | Residual | 2.320 | 1.523 |
|  | 19 |  | Pot:plot | <0.001 | 0.005 |
|  | 19 |  | Round | 0.001 | 0.023 |
|  | 19 |  | Plot | <0.001 | 0.002 |
|  | 19 |  | Residual | 0.001 | 0.029 |

**Fixed effects:**

| **response** | **year** | **factor** | **Coeff.** | **se** | **df** | **t** | **p** |
| --- | --- | --- | --- | --- | --- | --- | --- |
| **Mummy number** | 2018 | Intercept | 1.52 | 0.26 | 8 | 5.91 | 0.0004 |
|  |  | Number of aphids | 0.0007 | 0.0002 | 62 | 3.82 | 0.0003 |
|  |  | *H. defensa* infected versus ninfected | -0.43 | 0.20 | 70 | -2.11 | 0.0381 |
|  |  | *H. defensa* diversity | 0.06 | 0.21 | 69 | 0.26 | 0.7922 |
|  |  | *H. defensa* strain H15 | 0.06 | 0.27 | 68 | 0.22 | 0.8235 |
|  |  | *H. defensa* strain H76 | 0.24 | 0.26 | 67 | 0.91 | 0.3671 |
|  | 2019 | Intercept | 1.15 | 0.23 | 16 | 4.95 | 0.0001 |
|  |  | Number of aphids | 0.0009 | 0.0002 | 371 | 3.75 | 0.0002 |
|  |  | *H. defensa* infected versus ninfected | -0.19 | 0.14 | 22 | -1.37 | 0.1853 |
|  |  | *H. defensa* diversity | 0.10 | 0.15 | 21 | 0.65 | 0.5225 |
|  |  | *H. defensa* strain H15 | 0.38 | 0.17 | 19 | 2.23 | 0.0382 |
|  |  | *H. defensa* strain H76 | 0.17 | 0.17 | 19 | 1.00 | 0.3310 |
| **Mummification rate** | 2018 | Intercept | 0.52 | 0.07 | 68 | 7.12 | *<0.0001* |
|  |  | Number of aphids | -0.0001 | 0.00006 | 68 | -2.05 | 0.0446 |
|  |  | *H. defensa* infected versus ninfected | -0.03 | 0.08 | 71 | -0.43 | 0.6691 |
|  |  | *H. defensa* diversity | 0.07 | 0.08 | 70 | 0.85 | 0.3968 |
|  |  | *H. defensa* strain H15 | 0.15 | 0.10 | 68 | 1.53 | 0.1318 |
|  |  | *H. defensa* strain H76 | 0.23 | 0.10 | 68 | 2.36 | 0.0210 |
|  | 2019 | Intercept | 0.57 | 0.08 | 14 | 7.05 | *<0.0001* |
|  |  | Number of aphids | -0.0004 | 0.00008 | 282 | -4.92 | *<0.0001* |
|  |  | *H. defensa* infected versus ninfected | -0.06 | 0.05 | 24 | -1.12 | 0.2722 |
|  |  | *H. defensa* diversity | 0.08 | 0.05 | 24 | 1.60 | 0.1237 |
|  |  | *H. defensa* strain H15 | 0.12 | 0.06 | 20 | 2.16 | 0.0428 |
|  |  | *H. defensa* strain H76 | 0.06 | 0.06 | 21 | 1.05 | 0.3038 |
| **Number of hatched parasitoids** | 2018 | Intercept | 1.08 | 0.01 | 12 | 145.73 | *<0.0001* |
|  |  | Number of aphids | 0.00005 | 0.000006 | 50 | 7.68 | *<0.0001* |
|  |  | *H. defensa* infected versus ninfected | -0.02 | 0.01 | 70 | -2.69 | 0.0090 |
|  |  | *H. defensa* diversity | <0.01 | 0.01 | 69 | -0.27 | 0.7854 |
|  |  | *H. defensa* strain H15 | -0.01 | 0.01 | 68 | -0.88 | 0.3821 |
|  |  | *H. defensa* strain H76 | <0.01 | 0.01 | 67 | -0.59 | 0.5591 |
|  | 2019 | Intercept | -0.92 | 0.01 | 15 | -112.64 | *<0.0001* |
|  |  | Number of aphids | 0.00001 | 0.000002 | 26 | 5.08 | *<0.0001* |
|  |  | *H. defensa* infected versus ninfected | -0.01 | 0.01 | 22 | -1.37 | 0.1852 |
|  |  | *H. defensa* diversity | -0.01 | 0.01 | 21 | -1.43 | 0.1684 |
|  |  | *H. defensa* strain H15 | 0.01 | 0.01 | 19 | 2.41 | 0.0261 |
|  |  | *H. defensa* strain H76 | 0.02 | 0.01 | 19 | 2.59 | 0.0177 |
| **Number of aphids** | 2018 | Intercept | 3.94 | 0.74 | 3 | 5.30 | 0.0128 |
|  |  | *H. defensa* infected versus ninfected | -0.77 | 0.41 | 71 | -1.85 | 0.0680 |
|  |  | *H. defensa* diversity | -0.76 | 0.42 | 70 | -1.82 | 0.0733 |
|  |  | *H. defensa* strain H15 | 0.14 | 0.51 | 68 | 0.27 | 0.7863 |
|  |  | *H. defensa* strain H76 | -0.70 | 0.51 | 68 | -1.37 | 0.1737 |
|  | 2019 | Intercept | 1.73 | 0.37 | 19 | 4.74 | 0.0002 |
|  |  | *H. defensa* infected versus ninfected | -0.01 | 0.21 | 23 | -0.03 | 0.9790 |
|  |  | *H. defensa* diversity | -0.04 | 0.22 | 22 | -0.17 | 0.8694 |
|  |  | *H. defensa* strain H15 | 0.10 | 0.27 | 20 | 0.36 | 0.7189 |
|  |  | *H. defensa* strain H76 | -0.12 | 0.27 | 20 | -0.45 | 0.6589 |
| **Plant size** | 2018 | Intercept | 15.78 | 0.53 | 8 | 29.82 | *<0.0001* |
|  |  | Number of aphids | <0.01 | 0.00 | 58 | -1.16 | 0.2490 |
|  |  | *H. defensa* infected versus ninfected | 0.04 | 0.44 | 70 | 0.10 | 0.9244 |
|  |  | *H. defensa* diversity | -0.41 | 0.45 | 69 | -0.91 | 0.3637 |
|  |  | *H. defensa* strain H15 | -0.21 | 0.57 | 67 | -0.37 | 0.7157 |
|  |  | *H. defensa* strain H76 | -0.25 | 0.56 | 67 | -0.45 | 0.6554 |
|  | 2019 | Intercept | -0.30 | 0.01 | 20 | -40.74 | *<0.0001* |
|  |  | Number of aphids | <0.01 | <0.01 | 293 | -0.81 | 0.4201 |
|  |  | *H. defensa* infected versus ninfected | -0.01 | <0.01 | 23 | -1.53 | 0.1392 |
|  |  | *H. defensa* diversity | <0.01 | <0.01 | 22 | 0.65 | 0.5203 |
|  |  | *H. defensa* strain H15 | <0.01 | 0.01 | 20 | -0.07 | 0.9446 |
|  |  | *H. defensa* strain H76 | <0.01 | 0.01 | 20 | -0.30 | 0.7651 |

## **Table S3**

**Table S3: Summary of linear mixed models for different measures of parasitoid diversity (1 vs. 3 strains) using treatments as fixed effect and block (2018) or plant nested within block and round (2019) as random effects.** In order to obtain coefficients, we used three separate models, one including only *H. defensa* presence versus absence to estimate its effect (and aphid Number where appropriate), one additionally including, *H. defensa* diversity (3 versus 1 different haplotypes) to determine the effect of diversity, and one additionally including *H. defensa* strain (among strains; comparison against H402) to obtain estimates for the effect of strain identity. Residuals, Number of aphids, and Intercepts were obtained from the final model.

**Random effects:**

| **Response** | **Year** |  | **Group** | **Variance** | **sd** |
| --- | --- | --- | --- | --- | --- |
| **Species number** | 2018 |  | Block | <0.001 | <0.001 |
|  |  |  | Residual | 0.636 | 0.798 |
|  | 2019 |  | Plot | 369.155 | 19.213 |
|  |  |  | Residual | 257.228 | 16.038 |
| **Species number, primary parasitoids** | 2018 |  | Block | <0.001 | <0.001 |
|  |  |  | Residual | 0.434 | 0.659 |
|  | 2019 |  | Plot | <0.001 | 0.017 |
|  |  |  | Residual | 0.002 | 0.045 |
| **Shannon index** | 2018 |  | Block | 0.022 | 0.148 |
|  |  |  | Residual | 0.178 | 0.421 |
|  | 2019 |  | Plot | 0.167 | 0.409 |
|  |  |  | Residual | 0.057 | 0.238 |
| **Shannon index, primary parasitoids** | 2018 |  | Block | <0.001 | <0.001 |
|  |  |  | Residual | 0.106 | 0.326 |
|  | 2019 |  | Plot | 0.001 | 0.030 |
|  |  |  | Residual | 0.073 | 0.270 |
| **Rarefied species number** | 2018 |  | Block | 0.103 | 0.320 |
|  |  |  | Residual | 0.455 | 0.674 |
|  | 2019 |  | Plot | 2.509 | 1.584 |
|  |  |  | Residual | 1.467 | 1.211 |
| **Rarefied species number, primary parasitoids** | 2018 |  | Block | <0.001 | <0.001 |
|  |  |  | Residual | 0.348 | 0.590 |
|  | 2019 |  | Plot | 0.001 | 0.025 |
|  |  |  | Residual | 0.079 | 0.280 |
| **Rarefied Shannon index** | 2018 |  | Block | 0.018 | 0.136 |
|  |  |  | Residual | 0.093 | 0.305 |
|  | 2019 |  | Plot | 0.055 | 0.234 |
|  |  |  | Residual | 0.040 | 0.201 |
| **Rarefied Shannon index, primary parasitoids** | 2018 |  | Block | <0.001 | <0.001 |
|  |  |  | Residual | 0.106 | 0.325 |
|  | 2019 |  | Plot | <0.001 | <0.001 |
|  |  |  | Residual | 0.068 | 0.260 |

**Fixed effects:**

| **Response** | **Year** | **Factor** | **Coeff.** | **se** | **df** | **t** | **p** |
| --- | --- | --- | --- | --- | --- | --- | --- |
| **Species number** | 2018 | Intercept | 3.16 | 0.21 | 70 | 15.33 | *<0.0001* |
|  |  | *H. defensa* infected versus uninfected | -0.51 | 0.23 | 73 | -2.26 | 0.0266 |
|  |  | *H. defensa* diversity | 0.15 | 0.24 | 72 | 0.64 | 0.5270 |
|  |  | *H. defensa* strain H15 | -0.17 | 0.29 | 70 | -0.59 | 0.5592 |
|  |  | *H. defensa* strain H76 | -0.23 | 0.29 | 70 | -0.80 | 0.4293 |
|  | 2019 | Intercept | 56.70 | 10.22 | 10 | 5.55 | 0.0002 |
|  |  | *H. defensa* infected versus uninfected | -19.10 | 7.26 | 23 | -2.63 | 0.0149 |
|  |  | *H. defensa* diversity | 0.34 | 7.66 | 22 | 0.04 | 0.9654 |
|  |  | *H. defensa* strain H15 | -11.19 | 9.26 | 20 | -1.21 | 0.2408 |
|  |  | *H. defensa* strain H76 | 2.92 | 9.26 | 20 | 0.32 | 0.7555 |
| **Species number, primary parasitoids** | 2018 | Intercept | 1.47 | 0.17 | 70 | 8.61 | *<0.0001* |
|  |  | *H. defensa* infected versus uninfected | -0.39 | 0.19 | 73 | -2.08 | 0.0408 |
|  |  | *H. defensa* diversity | 0.04 | 0.19 | 72 | 0.23 | 0.8216 |
|  |  | *H. defensa* strain H15 | -0.05 | 0.24 | 70 | -0.20 | 0.8443 |
|  |  | *H. defensa* strain H76 | -0.13 | 0.24 | 70 | -0.54 | 0.5900 |
|  | 2019 | Intercept | 1.05 | 0.02 | 24 | 53.30 | *<0.0001* |
|  |  | *H. defensa* infected versus uninfected | -0.03 | 0.02 | 23 | -1.40 | 0.1735 |
|  |  | *H. defensa* diversity | <0.01 | 0.02 | 22 | 0.23 | 0.8174 |
|  |  | *H. defensa* strain H15 | 0.02 | 0.03 | 20 | 0.95 | 0.3539 |
|  |  | *H. defensa* strain H76 | 0.01 | 0.03 | 20 | 0.46 | 0.6518 |
| **Shannon index** | 2018 | Intercept | 1.14 | 0.14 | 8 | 8.28 | *<0.0001* |
|  |  | *H. defensa* infected versus uninfected | -0.27 | 0.12 | 71 | -2.20 | 0.0310 |
|  |  | *H. defensa* diversity | 0.13 | 0.12 | 70 | 1.07 | 0.2905 |
|  |  | *H. defensa* strain H15 | -0.10 | 0.15 | 68 | -0.63 | 0.5279 |
|  |  | *H. defensa* strain H76 | 0.01 | 0.15 | 68 | 0.04 | 0.9670 |
|  | 2019 | Intercept | 1.26 | 0.19 | 8 | 6.53 | 0.0002 |
|  |  | *H. defensa* infected versus uninfected | -0.09 | 0.12 | 23 | -0.74 | 0.4660 |
|  |  | *H. defensa* diversity | 0.04 | 0.13 | 22 | 0.32 | 0.7545 |
|  |  | *H. defensa* diversity.1 | 0.13 | 0.14 | 20 | 0.95 | 0.3540 |
|  |  | *H. defensa* strain H15 | -0.31 | 0.14 | 20 | -2.26 | 0.0349 |
|  |  | *H. defensa* strain H76 | 0.04 | 0.14 | 20 | 0.29 | 0.7755 |
| **Shannon index, primary parasitoids** | 2018 | Intercept | 0.30 | 0.08 | 70 | 3.59 | 0.0006 |
|  |  | *H. defensa* infected versus uninfected | -0.13 | 0.09 | 73 | -1.35 | 0.1802 |
|  |  | *H. defensa* diversity | -0.01 | 0.10 | 72 | -0.15 | 0.8806 |
|  |  | *H. defensa* strain H15 | -0.05 | 0.12 | 70 | -0.45 | 0.6537 |
|  |  | *H. defensa* strain H76 | -0.15 | 0.12 | 70 | -1.29 | 0.2021 |
|  | 2019 | Intercept | 0.25 | 0.11 | 25 | 2.24 | 0.0340 |
|  |  | *H. defensa* infected versus uninfected | -0.11 | 0.12 | 23 | -0.93 | 0.3628 |
|  |  | *H. defensa* diversity | 0.03 | 0.13 | 22 | 0.20 | 0.8407 |
|  |  | *H. defensa* strain H15 | 0.19 | 0.16 | 20 | 1.20 | 0.2450 |
|  |  | *H. defensa* strain H76 | 0.06 | 0.16 | 20 | 0.41 | 0.6842 |
| **Rarefied species number** | 2018 | Intercept | 2.85 | 0.25 | 5 | 11.21 | 0.0001 |
|  |  | *H. defensa* infected versus uninfected | -0.34 | 0.19 | 71 | -1.75 | 0.0843 |
|  |  | *H. defensa* diversity | 0.23 | 0.20 | 70 | 1.16 | 0.2489 |
|  |  | *H. defensa* strain H15 | -0.13 | 0.25 | 68 | -0.51 | 0.6085 |
|  |  | *H. defensa* strain H76 | 0.12 | 0.25 | 68 | 0.48 | 0.6314 |
|  | 2019 | Intercept | 6.08 | 0.81 | 10 | 7.47 | *<0.0001* |
|  |  | *H. defensa* infected versus uninfected | -0.34 | 0.61 | 23 | -0.56 | 0.5780 |
|  |  | *H. defensa* diversity | 0.27 | 0.64 | 22 | 0.43 | 0.6732 |
|  |  | *H. defensa* strain H15 | -1.75 | 0.70 | 20 | -2.50 | 0.0210 |
|  |  | *H. defensa* strain H76 | -0.22 | 0.70 | 20 | -0.32 | 0.7529 |
| **Rarefied species number, primary parasitoids** | 2018 | Intercept | 1.29 | 0.15 | 70 | 8.48 | *<0.0001* |
|  |  | *H. defensa* infected versus uninfected | -0.27 | 0.17 | 73 | -1.63 | 0.1072 |
|  |  | *H. defensa* diversity | 0.07 | 0.17 | 72 | 0.38 | 0.7066 |
|  |  | *H. defensa* strain H15 | -0.14 | 0.22 | 70 | -0.65 | 0.5207 |
|  |  | *H. defensa* strain H76 | -0.09 | 0.22 | 70 | -0.44 | 0.6645 |
|  | 2019 | Intercept | -0.74 | 0.11 | 25 | -6.41 | *<0.0001* |
|  |  | *H. defensa* infected versus uninfected | -0.12 | 0.12 | 23 | -0.99 | 0.3344 |
|  |  | *H. defensa* diversity | 0.02 | 0.13 | 22 | 0.18 | 0.8580 |
|  |  | *H. defensa* strain H15 | 0.20 | 0.16 | 20 | 1.21 | 0.2414 |
|  |  | *H. defensa* strain H76 | 0.08 | 0.16 | 20 | 0.52 | 0.6085 |
| **Rarefied Shannon index** | 2018 | Intercept | 0.79 | 0.11 | 6 | 7.07 | 0.0006 |
|  |  | *H. defensa* infected versus uninfected | -0.14 | 0.09 | 71 | -1.55 | 0.1261 |
|  |  | *H. defensa* diversity | 0.11 | 0.09 | 70 | 1.17 | 0.2461 |
|  |  | *H. defensa* strain H15 | -0.06 | 0.11 | 68 | -0.57 | 0.5713 |
|  |  | *H. defensa* strain H76 | 0.04 | 0.11 | 68 | 0.36 | 0.7196 |
|  | 2019 | Intercept | 0.76 | 0.13 | 11 | 6.03 | 0.0001 |
|  |  | *H. defensa* infected versus uninfected | -0.03 | 0.10 | 23 | -0.33 | 0.7475 |
|  |  | *H. defensa* diversity | 0.05 | 0.10 | 22 | 0.44 | 0.6651 |
|  |  | *H. defensa* strain H15 | -0.28 | 0.12 | 20 | -2.43 | 0.0247 |
|  |  | *H. defensa* strain H76 | -0.06 | 0.12 | 20 | -0.54 | 0.5918 |
| **Rarefied Shannon index, primary parasitoids** | 2018 | Intercept | 0.29 | 0.08 | 70 | 3.51 | 0.0008 |
|  |  | *H. defensa* infected versus uninfected | -0.12 | 0.09 | 73 | -1.33 | 0.1879 |
|  |  | *H. defensa* diversity | -0.01 | 0.10 | 72 | -0.11 | 0.9140 |
|  |  | *H. defensa* strain H15 | -0.05 | 0.12 | 70 | -0.46 | 0.6470 |
|  |  | *H. defensa* strain H76 | -0.15 | 0.12 | 70 | -1.29 | 0.2013 |
|  | 2019 | Intercept | 0.24 | 0.11 | 25 | 2.24 | 0.0341 |
|  |  | *H. defensa* infected versus uninfected | -0.11 | 0.12 | 23 | -0.92 | 0.3673 |
|  |  | *H. defensa* diversity | 0.03 | 0.12 | 27 | 0.25 | 0.8064 |
|  |  | *H. defensa* strain H15 | 0.19 | 0.15 | 25 | 1.26 | 0.2180 |
|  |  | *H. defensa* strain H76 | 0.07 | 0.15 | 25 | 0.48 | 0.6388 |
