# Supplementary material for: Bottom‐up effect of host protective symbionts on parasitoid diversity: Limited evidence from two field experiments
Source: J Anim Ecol. 2022 Jan 16;91(3):643–54. doi: 10.1111/1365-2656.13650 (PMC9306599; doi:10.1111/1365-2656.13650)
Supplement: Supplementary file 3 — Supplementary Material [file JANE-91-643-s003.docx]

# **Supplementary Methods**

## Test for migration

In order to test whether aphids migrated between plants in 2019, we collected aphids on three different days towards the end of the experiment and stored them at -20°C. Subsequently, we extracted aphid and symbiont DNA using high salt extractions (see Sunnucks and Hales 1994), but adapted to a 96 deep well plate format (Gouskov et al. 2016, Hafer-Hahmann and Vorburger 2020). This was followed with diagnostic PCRs (see Ferrari *et al.* 2011) to test for the presence of *H. defensa* with symbiont-specific primer pairs that amplify parts of the bacterial 16S rRNA gene. Additionally we amplified DNA of *Buchnera aphidicola* which as obligate symbiont of aphids should be present in all individuals and hence served as a control for successful DNA extraction. Samples that were negative for *B. aphidicola* were discarded. PCRs were multiplexed for both symbionts using forwards primer 16SA1 (AGAGTTTGATCMTGGCTCAG; Fukatsu and Nikoh 1998) and reverse primer Buch_R_CV2 (CCCCCACTTTRGTTTTTCAAC; Hafer-Hahmann and Vorburger 2020) for *B. aphidicola* and forwards primer 10F (AGTTTGATCATGGCTCAGATTG) and reverse primer T419R (AAATGGTATTCGCATTTATCG) for *H. defensa* (Ferrari et al. 2011). We only tested for the presenc eor absence of *H. defensa*, but not strain (among single strains) and discarded samples that were negative for *B. aphidicola* or did not show clear bands.

In the H- treatment, 9 out of 11 aphids tested (81%) were indeed negative for *H. defensa* (i.e. in the correct pot). Similarly, in the treatments that should have contained *H. defensa*, 43 out of 53 aphids (81%) indeed were positive for *H. defensa*.

We did not test for migration in 2019 since we assumed it to be less of an issue than in 2018 since plants only remained outside for two to three weeks.
