# Supplementary material for: Bottom‐up effect of host protective symbionts on parasitoid diversity: Limited evidence from two field experiments
Source: J Anim Ecol. 2022 Jan 16;91(3):643–54. doi: 10.1111/1365-2656.13650 (PMC9306599; doi:10.1111/1365-2656.13650)
Supplement: Supplementary file 4 — Supplementary Material [file JANE-91-643-s002.docx]

# Supplementary Results

## Effect of treatment on further variables

To obtain the number of ants and predators we calculated the total number of predators (i.e. ladybugs (Coccinellidae), lacewings (Chrysopidae), hoverflies (Syrphidae), and *Aphidoletes*) and ants irrespective of life stage and taxon. To analyze the total number of predators and ants, we used linear mixed effect models with round (2018) or pot identity nested in plot and round (2019) as random effects and added aphid number, H. defensa presence/ absence, H. defensa diversity, and H. defensa identity in this order as fixed effects, which was followed with type I analysis of variance (see main text). To analyze the number of parasitoids for primary parasitoid species of which we found more than 100 individuals in a year (only *A. Chaonia* in 2018; *A. Chaonia* and *L. Fabarum* in 2019), we used the same approach, but using total parasitoid counts over all rounds in 2019 and hence plot only as random effect in 2019.

Treatment never affected the number of predators (Figure S3A; 2018: *H. defensa* infected versus uninfected: F_1,67_=0.83; p=0.366; *H. defensa* diversity (1 vs. 3 strains): F_1,67_=0.45; p=0.506; *H. defensa* strain (among single strains): F_2,67_=0.33; p=0.717; 2019: *H. defensa* infected versus uninfected: F_1,20_=0.13; p=0.726; *H. defensa* diversity: F_1,20_=0.34; p=0.566; *H. defensa* strain: F_2,19_=1.30; p=0.294) or ants we counted (Figure S3B; 2018: *H. defensa* infected versus uninfected: F_1,67_=0.96; p=0.331; *H. defensa* diversity (1 vs. 3 strains): F_1,67_=0.09; p=0.765; *H. defensa* strain (among single strains): F_2,67_=0.03; p=0.969; 2019: *H. defensa* infected versus uninfected: F_1,19_=2.04; p=0.169; *H. defensa* diversity: F_1,19_=0.22; p=0.643; *H. defensa* strain: F_2,19_=0.81; p=0.461). Not surprisingly, the number of ants was strongly positively associated with the number of aphids in either year (2018: F_1,68_=24.32, p<0.001; 2019: F_1,370_=53.03, p<0.001. Predators showed a positive association with aphids only in 2019 (F_1,255_=45.03, p<0.001) but not in 2018 (F_1,68_=0.78, p=0.379).

Figure S4: Number of ants (A) and total number of predators (B). Error bars represent 95% CI, boxes represent SE. H-: *H. defensa* free aphids, H15: Aphids carrying *H. defensa* haplotype 15, H402: Aphids carrying *H. defensa* haplotype 402, H76: Aphids carrying *H. defensa* haplotype 76, Hmix: Aphids carrying *H. defensa* of different haplotypes. Please note that data is for three plants for 2018 and for a single plant in 2019.

*Aphidius chaonia* showed a marginally non-significant trend to occur more frequently in *H. defensa* free aphids in 2018 (F_1,68_=3.72; p=0.058), but not in 2019 (F_1,20_=1.52: p=0.233). It was never significantly associated with H*. defensa* diversity (2018: F_1,68_=3.72; p=0.058; 2019: F_1,20_=1.52: p=0.233) or strain identity (2018: F_2,68_=1.67; p=0.201; 2019: F_2,20_=0.02: p=0.877). Similarly, in 2019 *Lysiphabus fabarum* occured in higher number in *H. defensa*-free compared to *H. defensa* infected aphids (F_1,25_=4.28; p=0.049), but was not significantly affected by *H. defensa* diversity (F_1,25_=0.51; p=0.484) or strain (F_2,9_=0.67; p=0.544) in 2019 (Figure 3). In 2018 we found too few *L. Fabarum* to conduct any analysis.

## Effect of primary parasitoids on secondary parasitoids

To analyze associations between primary and secondary parasitoid community composition we used a distance based redundancy analysis (dbRDA) similar to described in the main text using the secondary parasitoid species as species matrix and primary parasitoid species matrix as environmental matrix.

Primary parasitoid communities significantly affected secondary parasitoid communities (Figure S4, 2018: F_9,53_=3.47, p=0.031; 2019: F_7,17_=28.16, p=0.005) explaining between 35.23% (2018) and 88.72% (2019) of variance. These associations seem to have been driven especially by *Lysiphlebus* spp. and *A.chaonia* (Table S5).

**Figure S5: First two axis from dbRDA to test for associations between primary (blue) and secondary parasitoids (red).** A: 2018; B: 2019. Only the first axis was significant (2018: F_9,53_=30.41, p=0.010; 2019: F_7,17_=216.60, p=0.005). The second axis was not (p>0.6). Please note that in order to improve readability, values for primary parasioids have been multiplied by 10. Primary parasitoids: Ach: *Aphelinus* *chaonia*, Ahu: *A. humilis*, Bac: *Binodoxys acalaphae*, Ban: *B. angelicae*, Epl: *Ephedrus plagiator*, Lgr: *Lipolexis gracilis*, Lca: *Lysiphlebus cardui*, Lfab: *L. fabarum*, Pva: *Praeon volucre*; Secondary parasitoids: All: *Alloxysta* spp., Asa: *Asaphes* spp., Den: *Dendrocerus* spp., Pap: *Prachyneuron aphidis*, Sap: *Syrphophagus aphidivorus.*

**Table S4: Outcome of permutation tests for dbRDA testing the significance of environmental variables (i.e. primary parasitoid species).** Significant p-values for the effect of treatment have been highlighted in bold, marginally significant ones in italic.

| **Species** | **2018** | | | **2019** | | |
| --- | --- | --- | --- | --- | --- | --- |
|  | **df** | **F** | **p** | **df** | **F** | **p** |
| *Aphelinus chaonia* | 1 | 3.23 | *0.061* | 1 | 74.84 | **0.004** |
| *Binodoxys acalephae* | 1 | 0.49 | 0.549 | 1 | 2.56 | *0.053* |
| *Binodoxys angelicae* | 1 | 0.38 | 0.399 | 1 | 41.64 | **0.037** |
| *Lysiphlebus cardui* | 1 | 4.13 | *0.068* | 1 | 66.02 | **0.012** |
| *Lysiphlebus fabarum* | 1 | 20.86 | **0.018** | 1 | 5.87 | *0.088* |
| *Praeon volucre* | 1 | 0.02 | 0.989 |  |  |  |
| *Aphelinus humilis* | 1 | 0.79 | 0.264 |  |  |  |
| *Ephedrus plagiator* | 1 | 0.14 | 0.865 | 1 | 4.76 | *0.081* |
| *Lipolexis gracilis* | 1 | 1.21 | 0.163 | 1 | 1.42 | 0.149 |
| Residual | 53 |  |  | 17 |  |  |
